# The top-cited systematic reviews/meta-analyses in tuberculosis research: A PRISMA-compliant systematic literature review and bibliometric analysis

**DOI:** 10.1097/MD.0000000000004822

**Published:** 2017-02-10

**Authors:** Yonggang Zhang, Jin Huang, Liang Du

**Affiliations:** a Department of Periodical Press, West China Hospital, Sichuan University, Chengdu, Sichuan, China; b Centre for Public Health, School of Medicine, Dentistry and Biomedical Sciences, Queen's University Belfast, Belfast, Northern Ireland, UK; c The Chinese Cochrane Centre, West China Hospital, Sichuan University, Chengdu, China.

**Keywords:** bibliometric, meta-analysis, systematic review, tuberculosis

## Abstract

**Background:**

The top-cited systematic reviews/meta-analyses in tuberculosis research have not been identified. The objective of this study was to identify the 100 top-cited systematic reviews/meta-analyses in tuberculosis research, and to understand factors resulting in highly cited works, and establish trends in systematic reviews/meta-analyses in tuberculosis research.

**Methods:**

The Web of Science Core Collection was searched for systematic reviews/meta-analyses on tuberculosis up to January 31, 2016. Articles were ranked by citation count and screened by 2 authors. The following information was collected and analyzed from each included study: citation of Web of Science Core Collection, author, country, year, journal, institution, page number, and reference number.

**Results:**

The 100 top-cited studies were cited from 54 to 662 times and were published between 1997 and 2014. Ten authors have more than 1 study as the first author and 10 authors have more than 1 study as corresponding author. The country with the most top-cited studies was USA (n = 26). The institutions with the largest number of the studies were McGill University in Canada (n = 18). The studies were published in 32 journals, whereas 12 were published in *PloS Medicine,* followed by *Lancet Infectious Diseases* (n = 11).

**Conclusions:**

Developed countries and high-impact journals may publish more top-cited systematic review/meta-analysis in tuberculosis research.

## Abstract 2

Supplemental Digital Content is available in the text

## 1 Introduction

Tuberculosis (TB) is a widespread, chronic infectious disease caused by *Mycobacterium tuberculosis*.^[1,2]^ TB generally affects the lungs, but can also affect other parts of the body.^[3]^ One-third of the world's population is thought to have been infected with *M tuberculosis.*^[4]^ The World Health Organization (WHO) reported that 9.6 million people fell ill with TB and 1.5 million died from TB in 2014.^[5]^ It is urgent to study the etiology, prevention, diagnostic, and treatment of TB.^[6]^

Although there are many studies in the same topic in TB researches, however, the results may be inconclusive.^[7]^ Since the using of systematic review/meta-analysis, studies in the same topic can be pooled and give more precise results.^[8]^ Systematic review/meta-analysis tries to appraise, select, and synthesize all high-quality research evidence relevant to a certain question.^[9]^ It aims at seeking patterns among study results and agreement or disagreement among those results.^[10,11]^ Up to now, there have been many systematic reviews/meta-analyses in the field of TB research,^[12–15]^ and these studies have contributed too many fields of controlling TB. However, the performance, productivity, and impact of these studies are still unknown. To evaluate the current body of these studies, detecting trends is urgent for future TB researches. Bibliometric analysis is the most important method.^[16,17]^

One of the most useful methodologies of bibliometric analysis is citation analysis, which will examine a network of published articles to assess the individual article's impact and influence on its field.^[18,19]^ Many journals have published the most cited articles in their given field, such as radiology,^[17]^ diabetes,^[20]^ and so on. Previously, we reported the most cited studies in the field of TB^[16]^; we felt that study was still needed to be strength because we did not report the results in the specific filed. Considering the systematic reviews/meta-analyses contributing in many ways of TB, thus, we performed the current study. The purpose of this study was to identify and analyze the 100 most cited systematic reviews/meta-analyses in TB research across all peer-reviewed scientific journals. This bibliometric analysis reflects the most influential systematic reviews/meta-analyses to date in TB field and helps to identify future trends in TB research.

## 2 Materials and methods

This is a bibliometric analysis, so ethical approval was not required for the study. The study was conducted according to the Preferred Reporting Items for Systematic Reviews and Meta-analysis (PRISMA) statement.^[21]^

### 2.1 Study search

We performed a bibliometric analysis of the most highly cited systematic reviews/meta-analyses in the field of TB on January 31, 2016. All journals in Web of Science Core Collection were eligible for inclusion. The following search terms were used: “tuberculosis” or “TB” or “tuberculo∗” and “systematic review” or “meta-analysis.” The search results were sorted by number of citations.

### 2.2 Inclusion and exclusion criteria

The following inclusion criteria were used: the study should be a systematic review or meta-analysis in the field of TB research; the studies should be articles, reviews, editorials, or research letters. The exclusion criteria were as follows: abstracts, correction; study that mentioned the word “tuberculosis,” but not deal with TB.

### 2.3 Data extraction and analysis

The 100 top-cited systematic reviews/meta-analyses were identified based on the citations. Two authors independently extracted the following information from each study: number of citations, authors, corresponding authors (contact authors), authors’ address, Journal, year, country of origin, number of references, and number of page. We analyzed the relationship between citations and distribution of authors, countries, institutions, years, and journals. The country was determined on the basis of the address of the first author, whereas the institution was determined on the basis of the address of corresponding author. If the first author and corresponding author had more than 1 address, the first address was used for analysis. Any disagreement was resolved by discussion or decided by the third author.

## 3 Result

### 3.1 The main characteristics of the included studies

The Supplement Table 1 shows characteristics of the 100 top-cited TB systematic reviews/meta-analyses in descending order. The number of citation of these studies varies from 54 to 662, with a total citation of 13,090. The most cited study with a total of 662 citations was about T-cell-based assays for the diagnosis of latent TB infection,^[22]^ and this study was published in 2008 by Pai et al in *Annals of Internal Medicine.* The second study was a meta-analysis on new tests for the diagnosis of latent TB infection, published in *Annals of Internal Medicine* in 2007 by Menzies et al^[23]^ The third most cited study with 559 citations,^[24]^ with the title “*Interferonn assays-gamma in the immunodiagnosis of tuberculosis: a systematic review*,” was published in *Lancet Infectious Diseases* in 2004 by Pai et al. The reference numbers for the included studies were from 10 to 470, with an average of 69 references. The page numbers for the included studies were from 4 to 171, with an average of 13 pages.

### 3.2 Distribution of authors

Forty-one studies had less than 5 authors, and 11 studies had more than 10 authors. In quantitative terms, 10 authors have more than 1 study in 100 top-cited articles as the first authors, and 10 authors have more than 1 study as corresponding author (Table 1). The author with most studies as corresponding author was Pai (n = 17).

**Table 1:**
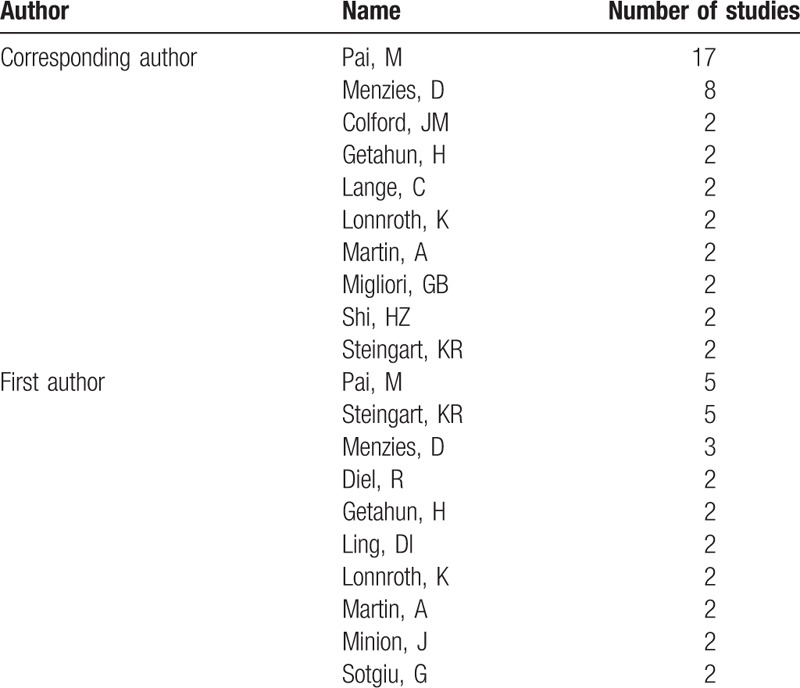
Authors with more than one study as first or corresponding authors included in the 100 top-cited studies.

### 3.3 Distribution of countries

The 100 top-cited TB systematic reviews/meta-analyses were from 21 countries (Australia, Belgium, Brazil, Canada, Chile, China, England, France, Germany, Italy, Mexico, Nepal, The Netherlands, Norway, Saudi Arabia, South Africa, Spain, Switzerland, Uganda, USA, and Wales). The country with the most top-cited systematic review/meta-analysis was USA with 26 studies, followed by Canada with 22 studies, Switzerland with 22 studies, England with 8 studies, and China with 6 studies. The country with the most citations was Canada with 3648 citations, followed by USA with 3494 citations. The country with most average citations was Norway with 242 citations, followed by Canada with 166 citations (Table 2).

**Table 2:**
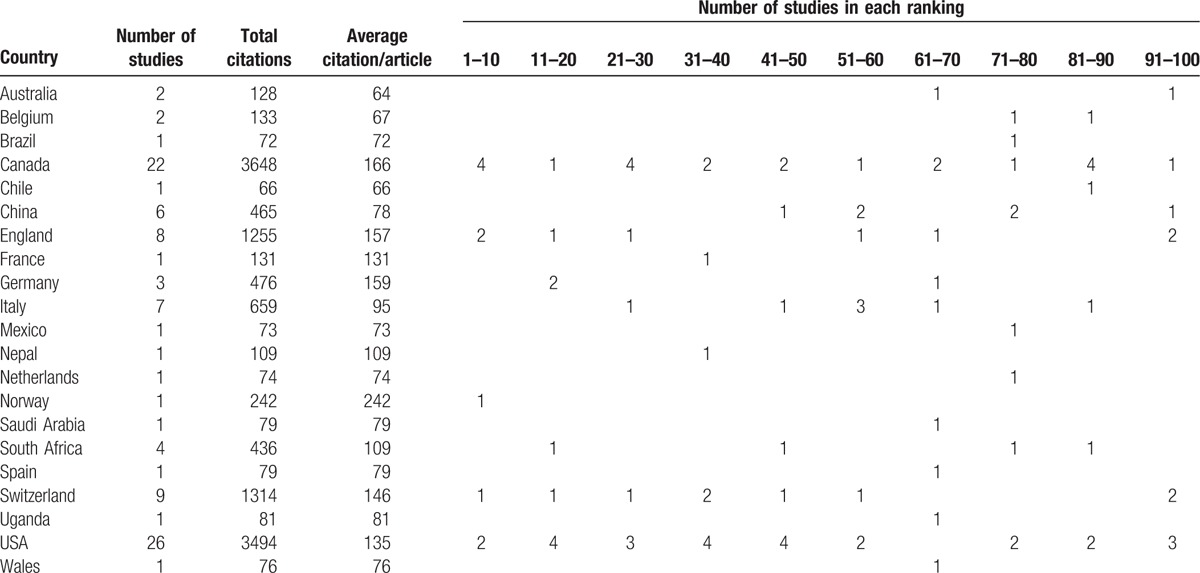
Country of origin of the 100 top-cited studies (based on country of first author).

### 3.4 Distribution of institutions

A total of 13 institutions with more than 2 studies were included (Table 3). The institutions with the largest number of the articles were McGill University in Canada (n = 18) and University of California, Berkeley in USA (n = 7), followed by WHO (n = 6), Harvard University (n = 4).

**Table 3:**
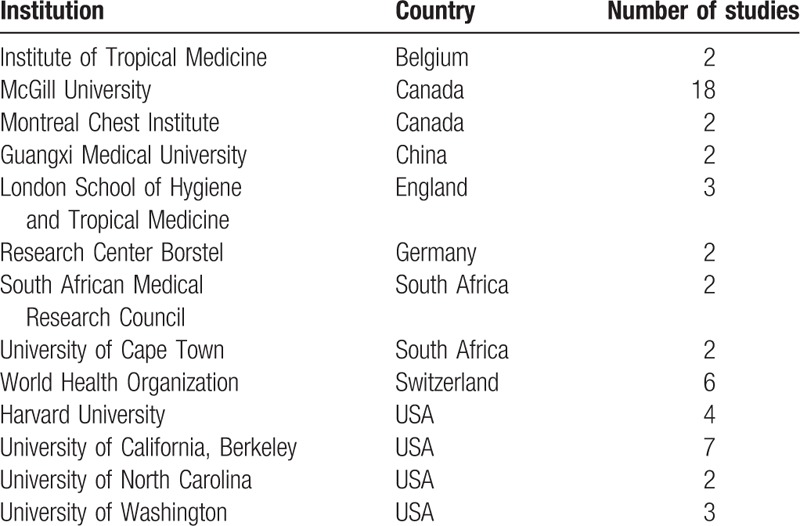
Institutions with at least 2 studies based on the institution of the corresponding authors included in the 100 top-cited studies.

### 3.5 Distribution of published years

Year distribution of the 100 top-cited studies is list in Table 4. These studies were published from 1997 to 2014. The year with most studies was 2008 with 15 studies, followed by 2007 with 13 studies. The year with most citations was 2008 with 2439 citations, followed by 2006 with 1348 citations. The year with most average citation was 2002 with 323, followed by 2004 with 251.

**Table 4:**
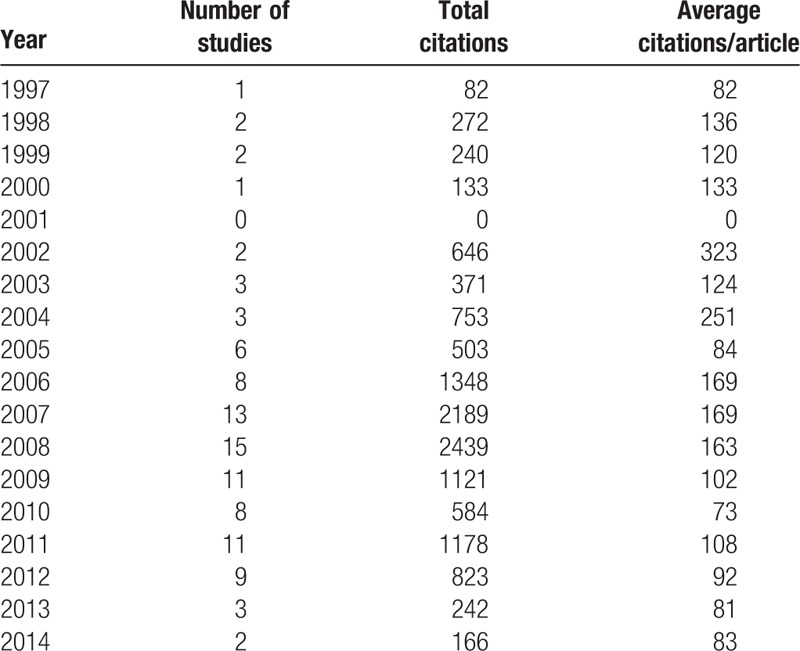
Distribution by year of publication of the 100 top-cited studies.

### 3.6 Distribution of published journals

The 100 studies were published in 32 journals (Table 5). The journal with the largest number of the articles cited was *PloS Medicine* (n = 12), followed by *Lancet Infectious Diseases* (n = 11) and *International Journal of Tuberculosis and Lung Disease* (n = 10). The journal with most citations was *Lancet Infectious Diseases* with 1962 citations, followed by *PloS Medicine* with 1522 citations and *Annals of Internal Medicine* with 1319 citations. The journal with the highest average number of citations per article was *Annals of Internal Medicine* with 440 citations, followed by *Lancet* with 240 citations.

**Table 5:**
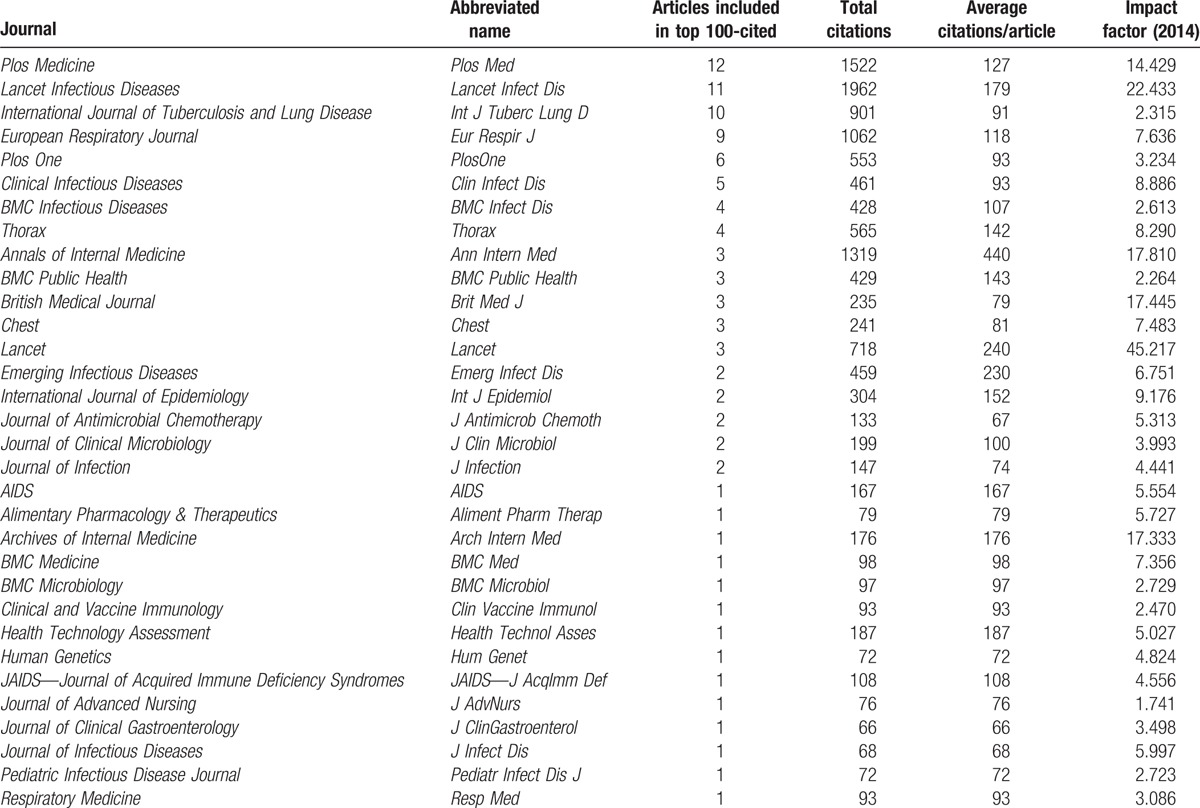
Journals in which the 100 top-cited studies were published.

## 4 Discussion

In recent years, with the prevalence of HIV^[25]^ and drug-resistant MTB,^[26]^ TB has become a dire threat to human health. TB research is also one of the most important research topics of medical researchers. Systematic review/meta-analysis in the field of TB provides many evidence for prevention, diagnosis, and treatment of TB. However, no citation analysis focused on this field; thus, we performed the current study to identify the 100 top-cited TB systematic reviews/meta-analyses and to analyze their principal characteristics.

The number of citations for the 100 top-cited systematic reviews/meta-analyses ranged from 54 to 662, which is far more less that the number of citation of 100 top-cited studies on TB (366–4443), which suggest that the popularization of systematic reviews/meta-analyses remains to be improved. In our study, 100 top-cited studies were published from 1997 to 2014, except 2001. Most of the studies were published in 2000, which is consistent with the development of the methods of systematic review/meta-analysis.

The top 3 ranked studies are all about the T-cell-based assays for diagnosis of TB,^[22–24]^ which partly suggested the diagnosis of TB, especially for latent TB infection, is still the most difficult and important research topic for TB. The number 1 ranked study is a systematic review about the T-cell-based assays for the diagnosis of latent TB infection. Based on the records in the Web of Science Core Collection, this study has been cited in many other research areas, including rheumatology, pediatrics, gastroenterology, dermatology, surgery, and so on. It has also been cited in 7 languages, including English, French, Portuguese, Italian, Spanish, German, and Polish, which indicated the study had an effect worldwide.

Consistent with similar studies, almost half included articles were from USA and Canada.^[16]^ The institutions with largest numbers of the articles were McGill University in Canada and University of California, Berkeley, in USA. It closely related to the influence and scientific output of the field of TB in Canada and USA. Some developing counties, such as China, Mexico, and Nepal, also published some top cited systematic review/meta-analysis. However, no study was from India, the country with heaviest burden of TB. In addition, no study from developing country ranked in the top 10. The only 1 study from a developing country ranked in top 20 cited studies^[27]^ was an international cooperative study between South Africa, England, and Norway. The developed countries may pay more attention on the topic and have more funds.

The results from our analysis indicated that the most highly cited systematic reviews/meta-analyses were published in journals related to infectious and respiratory diseases, such as *International Journal of Tuberculosis and Lung Disease*, *Lancet Infectious Diseases*, and *European Respiratory Journal*. Comprehensive medical periodicals have published articles among top 100-cited as well, like *PloS Medicine*, *PloS One*, and *BMJ*. We have to mention the *International Journal of Tuberculosis and Lung Disease.* This journal has a relative lower impact factor than most of the included journals. However, a total of 10 articles published in the journal were included.^[28–37]^ They ranked 21st,^[28]^ 31st,^[29]^ 44th,^[30]^ 54th,^[31]^ 59th,^[32]^ 76th,^[33]^ 77th,^[34]^ 80th,^[35]^ 97th,^[36]^ and 99th.^[37]^ They all have been cited more than 50 times. Because the history of research tells us that milestone papers can be rejected when they first submitted;^[20,38]^ thus, to reveal some gold papers, the editors, reviewers should keep guard on quality and be more open to the arriving manuscripts.^[20,38]^

There are several limitations of this study. First, the current study was only based on journal studies from Web of Science database. Web of Science does not index all journals and there are other databases available for citation analysis (such as Scopus and Google Scholar). Therefore, there may be some missed literature. Thus, our results should be explained with caution. Second, when we analyzed the origin country, our study was based on the institution address of corresponding author. If the author changes the address, there might be statistic bias. Third, there may be many factors that influence the number of citations for one study. We did not analyze self-citation, citations in textbooks, lectures, and journals not included by Web of Science Core Collection.^[20]^ In addition, some authors might cite studies in some journals in which they hoped to publish their research.^[20]^ Fourth, there might be some important and influential articles with lower citation numbers that were not included. After all, Web of Science Core Collection includes the most influential studies worldwide with short updating period, our study have the enormous advantage.

The aim of the current study was to analyze the-top cited systematic review/meta-analysis in TB research, which is partly different from other bibliometric studies in the field of radiology and diabetes. First, systematic review/meta-analysis analyzes disagreement conclusions and gives conclusive results, which will impact longer than original studies in clinical practice. Second, the average citations included in current study might increase quicker than those in our previous study. Third, the summary of the most conclusive research in TB research field may have more implication for the future clinical practices and research work.

## 5 Conclusions

In conclusion, the current study is the first bibliometric assessment of the TB systematic reviews/meta-analyses. Developed countries and high-impact journals may publish more top cited systematic review/meta-analysis in the field of TB.

## Supplementary Material

Supplemental Digital Content
