# Supplementary material for: The top-cited systematic reviews/meta-analyses in tuberculosis research: A PRISMA-compliant systematic literature review and bibliometric analysis
Source: Medicine (Baltimore). 2017 Feb 10;96(6):e4822. doi: 10.1097/MD.0000000000004822 (PMC5312977; doi:10.1097/MD.0000000000004822)
Supplement: Supplemental Digital Content [file medi-96-e4822-s001.docx]

**Supplement table 1 The top 100-cited systematic review/meta-analysis in tuberculosis research**

| Ranking | Study | Citation number |
| --- | --- | --- |
| 1 | Pai M, Zwerling A, Menzies D. Systematic review: T-cell-based assays for the diagnosis of latent tuberculosis infection: an update. Ann Intern Med. 2008;149(3):177-84. | 662 |
| 2 | Menzies D, Pai M, Comstock G. Meta-analysis: new tests for the diagnosis of latent tuberculosis infection: areas of uncertainty and recommendations for research. Ann Intern Med. 2007;146(5):340-54. | 568 |
| 3 | Pai M, Riley LW, Colford JM, Jr. Interferon-gamma assays in the immunodiagnosis of tuberculosis: a systematic review. Lancet Infect Dis. 2004;4(12):761-76. | 559 |
| 4 | Glynn JR, Whiteley J, Bifani PJ, Kremer K, van Soolingen D. Worldwide occurrence of Beijing/W strains of Mycobacterium tuberculosis: a systematic review. Emerg Infect Dis. 2002;8(8):843-9. | 373 |
| 5 | Trunz BB, Fine P, Dye C. Effect of BCG vaccination on childhood tuberculous meningitis and miliary tuberculosis worldwide: a meta-analysis and assessment of cost-effectiveness. Lancet. 2006;367(9517):1173-80. | 371 |
| 6 | Wang L, Turner MO, Elwood RK, Schulzer M, FitzGerald JM. A meta-analysis of the effect of Bacille Calmette Guerin vaccination on tuberculin skin test measurements. Thorax. 2002;57(9):804-9. | 273 |
| 7 | Jeon CY, Murray MB. Diabetes mellitus increases the risk of active tuberculosis: a systematic review of 13 observational studies. PLoS Med. 2008;5(7):e152. | 271 |
| 8 | Steingart KR, Henry M, Ng V, Hopewell PC, Ramsay A, Cunningham J, et al. Fluorescence versus conventional sputum smear microscopy for tuberculosis: a systematic review. Lancet Infect Dis. 2006;6(9):570-81. | 265 |
| 9 | Nnoaham KE, Clarke A. Low serum vitamin D levels and tuberculosis: a systematic review and meta-analysis. Int J Epidemiol. 2008;37(1):113-9. | 244 |
| 10 | Storla DG, Yimer S, Bjune GA. A systematic review of delay in the diagnosis and treatment of tuberculosis. BMC Public Health. 2008;8:15. | 242 |
| 11 | Getahun H, Harrington M, O'Brien R, Nunn P. Diagnosis of smear-negative pulmonary tuberculosis in people with HIV infection or AIDS in resource-constrained settings: informing urgent policy changes. Lancet. 2007;369(9578):2042-9. | 236 |
| 12 | Orenstein EW, Basu S, Shah NS, Andrews JR, Friedland GH, Moll AP, et al. Treatment outcomes among patients with multidrug-resistant tuberculosis: systematic review and meta-analysis. Lancet Infect Dis. 2009;9(3):153-61. | 219 |
| 13 | Diel R, Goletti D, Ferrara G, Bothamley G, Cirillo D, Kampmann B, et al. Interferon-gamma release assays for the diagnosis of latent Mycobacterium tuberculosis infection: a systematic review and meta-analysis. Eur Respir J. 2011;37(1):88-99. | 213 |
| 14 | Steingart KR, Ng V, Henry M, Hopewell PC, Ramsay A, Cunningham J, et al. Sputum processing methods to improve the sensitivity of smear microscopy for tuberculosis: a systematic review. Lancet Infect Dis. 2006;6(10):664-74. | 208 |
| 15 | Lin HH, Ezzati M, Murray M. Tobacco smoke, indoor air pollution and tuberculosis: a systematic review and meta-analysis. PLoS Med. 2007;4(1):e20. | 199 |
| 16 | Munro SA, Lewin SA, Smith HJ, Engel ME, Fretheim A, Volmink J. Patient adherence to tuberculosis treatment: a systematic review of qualitative research. PLoS Med. 2007;4(7):e238. | 197 |
| 17 | Dinnes J, Deeks J, Kunst H, Gibson A, Cummins E, Waugh N, et al. A systematic review of rapid diagnostic tests for the detection of tuberculosis infection. Health Technol Assess. 2007;11(3):1-196. | 187 |
| 18 | Sester M, Sotgiu G, Lange C, Giehl C, Girardi E, Migliori GB, et al. Interferon-gamma release assays for the diagnosis of active tuberculosis: a systematic review and meta-analysis. Eur Respir J. 2011;37(1):100-11. | 185 |
| 19 | Pai M, Flores LL, Pai N, Hubbard A, Riley LW, Colford JM, Jr. Diagnostic accuracy of nucleic acid amplification tests for tuberculous meningitis: a systematic review and meta-analysis. Lancet Infect Dis. 2003;3(10):633-43. | 176 |
| 20 | Bates MN, Khalakdina A, Pai M, Chang L, Lessa F, Smith KR. Risk of tuberculosis from exposure to tobacco smoke: a systematic review and meta-analysis. Arch Intern Med. 2007;167(4):335-42. | 176 |
| 21 | Sterne JA, Rodrigues LC, Guedes IN. Does the efficacy of BCG decline with time since vaccination? Int J Tuberc Lung Dis. 1998;2(3):200-7. | 173 |
| 22 | Ling DI, Zwerling AA, Pai M. GenoType MTBDR assays for the diagnosis of multidrug-resistant tuberculosis: a meta-analysis. Eur Respir J. 2008;32(5):1165-74. | 172 |
| 23 | Bucher HC, Griffith LE, Guyatt GH, Sudre P, Naef M, Sendi P, et al. Isoniazid prophylaxis for tuberculosis in HIV infection: a meta-analysis of randomized controlled trials. AIDS. 1999;13(4):501-7. | 167 |
| 24 | Rangaka MX, Wilkinson KA, Glynn JR, Ling D, Menzies D, Mwansa-Kambafwile J, et al. Predictive value of interferon-gamma release assays for incident active tuberculosis: a systematic review and meta-analysis. Lancet Infect Dis. 2012;12(1):45-55. | 153 |
| 25 | Johnston JC, Shahidi NC, Sadatsafavi M, Fitzgerald JM. Treatment outcomes of multidrug-resistant tuberculosis: a systematic review and meta-analysis. PLoS One. 2009;4(9):e6914. | 152 |
| 26 | Faustini A, Hall AJ, Perucci CA. Risk factors for multidrug resistant tuberculosis in Europe: a systematic review. Thorax. 2006;61(2):158-63. | 151 |
| 27 | Morrison J, Pai M, Hopewell PC. Tuberculosis and latent tuberculosis infection in close contacts of people with pulmonary tuberculosis in low-income and middle-income countries: a systematic review and meta-analysis. Lancet Infect Dis. 2008;8(6):359-68. | 136 |
| 28 | Morgan M, Kalantri S, Flores L, Pai M. A commercial line probe assay for the rapid detection of rifampicin resistance in Mycobacterium tuberculosis: a systematic review and meta-analysis. BMC Infect Dis. 2005;5:62. | 134 |
| 29 | Brewer TF. Preventing tuberculosis with bacillus Calmette-Guerin vaccine: a meta-analysis of the literature. Clin Infect Dis. 2000;31 Suppl 3:S64-7. | 133 |
| 30 | Joshi R, Reingold AL, Menzies D, Pai M. Tuberculosis among health-care workers in low- and middle-income countries: a systematic review. PLoS Med. 2006;3(12):e494. | 132 |
| 31 | Slama K, Chiang CY, Enarson DA, Hassmiller K, Fanning A, Gupta P, et al. Tobacco and tuberculosis: a qualitative systematic review and meta-analysis. Int J Tuberc Lung Dis. 2007;11(10):1049-61. | 131 |
| 32 | Getahun H, Kittikraisak W, Heilig CM, Corbett EL, Ayles H, Cain KP, et al. Development of a standardized screening rule for tuberculosis in people living with HIV in resource-constrained settings: individual participant data meta-analysis of observational studies. PLoS Med. 2011;8(1):e1000391. | 126 |
| 33 | Ahuja SD, Ashkin D, Avendano M, Banerjee R, Bauer M, Bayona JN, et al. Multidrug resistant pulmonary tuberculosis treatment regimens and patient outcomes: an individual patient data meta-analysis of 9,153 patients. PLoS Med. 2012;9(8):e1001300. | 126 |
| 34 | Jacobson KR, Tierney DB, Jeon CY, Mitnick CD, Murray MB. Treatment outcomes among patients with extensively drug-resistant tuberculosis: systematic review and meta-analysis. Clin Infect Dis. 2010;51(1):6-14. | 119 |
| 35 | Lonnroth K, Williams BG, Stadlin S, Jaramillo E, Dye C. Alcohol use as a risk factor for tuberculosis - a systematic review. BMC Public Health. 2008;8:289. | 114 |
| 36 | Murray CJ, Ortblad KF, Guinovart C, Lim SS, Wolock TM, Roberts DA, et al. Global, regional, and national incidence and mortality for HIV, tuberculosis, and malaria during 1990-2013: a systematic analysis for the Global Burden of Disease Study 2013. Lancet. 2014;384(9947):1005-70. | 111 |
| 37 | Sarmiento OL, Weigle KA, Alexander J, Weber DJ, Miller WC. Assessment by meta-analysis of PCR for diagnosis of smear-negative pulmonary tuberculosis. J Clin Microbiol. 2003;41(7):3233-40. | 109 |
| 38 | Sreeramareddy CT, Panduru KV, Menten J, Van den Ende J. Time delays in diagnosis of pulmonary tuberculosis: a systematic review of literature. BMC Infect Dis. 2009;9:91. | 109 |
| 39 | Steingart KR, Henry M, Laal S, Hopewell PC, Ramsay A, Menzies D, et al. Commercial serological antibody detection tests for the diagnosis of pulmonary tuberculosis: a systematic review. PLoS Med. 2007;4(6):e202. | 108 |
| 40 | Cattamanchi A, Smith R, Steingart KR, Metcalfe JZ, Date A, Coleman C, et al. Interferon-gamma release assays for the diagnosis of latent tuberculosis infection in HIV-infected individuals: a systematic review and meta-analysis. J Acquir Immune Defic Syndr. 2011;56(3):230-8. | 108 |
| 41 | van Zyl-Smit RN, Zwerling A, Dheda K, Pai M. Within-subject variability of interferon-g assay results for tuberculosis and boosting effect of tuberculin skin testing: a systematic review. PLoS One. 2009;4(12):e8517. | 105 |
| 42 | Pai M, Flores LL, Hubbard A, Riley LW, Colford JM, Jr. Nucleic acid amplification tests in the diagnosis of tuberculous pleuritis: a systematic review and meta-analysis. BMC Infect Dis. 2004;4:6. | 104 |
| 43 | Ling DI, Flores LL, Riley LW, Pai M. Commercial nucleic-acid amplification tests for diagnosis of pulmonary tuberculosis in respiratory specimens: meta-analysis and meta-regression. PLoS One. 2008;3(2):e1536. | 102 |
| 44 | Mandalakas AM, Detjen AK, Hesseling AC, Benedetti A, Menzies D. Interferon-gamma release assays and childhood tuberculosis: systematic review and meta-analysis. Int J Tuberc Lung Dis. 2011;15(8):1018-32. | 101 |
| 45 | Wilkinson D, Squire SB, Garner P. Effect of preventive treatment for tuberculosis in adults infected with HIV: systematic review of randomised placebo controlled trials. BMJ. 1998;317(7159):625-9. | 99 |
| 46 | Baker MA, Harries AD, Jeon CY, Hart JE, Kapur A, Lonnroth K, et al. The impact of diabetes on tuberculosis treatment outcomes: a systematic review. BMC Med. 2011;9:81. | 98 |
| 47 | Flores LL, Pai M, Colford JM, Jr., Riley LW. In-house nucleic acid amplification tests for the detection of Mycobacterium tuberculosis in sputum specimens: meta-analysis and meta-regression. BMC Microbiol. 2005;5:55. | 97 |
| 48 | Suthar AB, Lawn SD, del Amo J, Getahun H, Dye C, Sculier D, et al. Antiretroviral therapy for prevention of tuberculosis in adults with HIV: a systematic review and meta-analysis. PLoS Med. 2012;9(7):e1001270. | 93 |
| 49 | Liang QL, Shi HZ, Wang K, Qin SM, Qin XJ. Diagnostic accuracy of adenosine deaminase in tuberculous pleurisy: a meta-analysis. Respir Med. 2008;102(5):744-54. | 93 |
| 50 | Sotgiu G, Centis R, D'Ambrosio L, Alffenaar JW, Anger HA, Caminero JA, et al. Efficacy, safety and tolerability of linezolid containing regimens in treating MDR-TB and XDR-TB: systematic review and meta-analysis. Eur Respir J. 2012;40(6):1430-42. | 93 |
| 51 | Sotgiu G, Ferrara G, Matteelli A, Richardson MD, Centis R, Ruesch-Gerdes S, et al. Epidemiology and clinical management of XDR-TB: a systematic review by TBNET. Eur Respir J. 2009;33(4):871-81. | 93 |
| 52 | Steingart KR, Dendukuri N, Henry M, Schiller I, Nahid P, Hopewell PC, et al. Performance of purified antigens for serodiagnosis of pulmonary tuberculosis: a meta-analysis. Clin Vaccine Immunol. 2009;16(2):260-76. | 93 |
| 53 | Falzon D, Gandhi N, Migliori GB, Sotgiu G, Cox HS, Holtz TH, et al. Resistance to fluoroquinolones and second-line injectable drugs: impact on multidrug-resistant TB outcomes. Eur Respir J. 2013;42(1):156-68. | 91 |
| 54 | Mase SR, Ramsay A, Ng V, Henry M, Hopewell PC, Cunningham J, et al. Yield of serial sputum specimen examinations in the diagnosis of pulmonary tuberculosis: a systematic review. Int J Tuberc Lung Dis. 2007;11(5):485-95. | 91 |
| 55 | Jiang J, Shi HZ, Liang QL, Qin SM, Qin XJ. Diagnostic value of interferon-gamma in tuberculous pleurisy: a metaanalysis. Chest. 2007;131(4):1133-41. | 90 |
| 56 | Cruciani M, Scarparo C, Malena M, Bosco O, Serpelloni G, Mengoli C. Meta-analysis of BACTEC MGIT 960 and BACTEC 460 TB, with or without solid media, for detection of mycobacteria. J Clin Microbiol. 2004;42(5):2321-5. | 90 |
| 57 | Lew W, Pai M, Oxlade O, Martin D, Menzies D. Initial drug resistance and tuberculosis treatment outcomes: systematic review and meta-analysis. Ann Intern Med. 2008;149(2):123-34. | 89 |
| 58 | Chang K, Lu W, Wang J, Zhang K, Jia S, Li F, et al. Rapid and effective diagnosis of tuberculosis and rifampicin resistance with Xpert MTB/RIF assay: a meta-analysis. J Infect. 2012;64(6):580-8. | 88 |
| 59 | Greco S, Girardi E, Masciangelo R, Capoccetta GB, Saltini C. Adenosine deaminase and interferon gamma measurements for the diagnosis of tuberculous pleurisy: a meta-analysis. Int J Tuberc Lung Dis. 2003;7(8):777-86. | 86 |
| 60 | Balcells ME, Thomas SL, Godfrey-Faussett P, Grant AD. Isoniazid preventive therapy and risk for resistant tuberculosis. Emerg Infect Dis. 2006;12(5):744-51. | 86 |
| 61 | Volmink J, Garner P. Systematic review of randomised controlled trials of strategies to promote adherence to tuberculosis treatment. BMJ. 1997;315(7120):1403-6. | 82 |
| 62 | Bwanga F, Hoffner S, Haile M, Joloba ML. Direct susceptibility testing for multi drug resistant tuberculosis: a meta-analysis. BMC Infect Dis. 2009;9:67. | 81 |
| 63 | Zwerling A, van den Hof S, Scholten J, Cobelens F, Menzies D, Pai M. Interferon-gamma release assays for tuberculosis screening of healthcare workers: a systematic review. Thorax. 2012;67(1):62-70. | 80 |
| 64 | Ena J, Valls V. Short-course therapy with rifampin plus isoniazid, compared with standard therapy with isoniazid, for latent tuberculosis infection: a meta-analysis. Clin Infect Dis. 2005;40(5):670-6. | 79 |
| 65 | Sanai FM, Bzeizi KI. Systematic review: tuberculous peritonitis--presenting features, diagnostic strategies and treatment. Aliment Pharmacol Ther. 2005;22(8):685-700. | 79 |
| 66 | Diel R, Loddenkemper R, Nienhaus A. Predictive value of interferon-gamma release assays and tuberculin skin testing for progression from latent TB infection to disease state: a meta-analysis. Chest. 2012;142(1):63-75. | 78 |
| 67 | Migliori GB, Sotgiu G, Gandhi NR, Falzon D, DeRiemer K, Centis R, et al. Drug resistance beyond extensively drug-resistant tuberculosis: individual patient data meta-analysis. Eur Respir J. 2013;42(1):169-79. | 77 |
| 68 | Noyes J, Popay J. Directly observed therapy and tuberculosis: how can a systematic review of qualitative research contribute to improving services? A qualitative meta-synthesis. J Adv Nurs. 2007;57(3):227-43. | 76 |
| 69 | Khan FA, Minion J, Pai M, Royce S, Burman W, Harries AD, et al. Treatment of active tuberculosis in HIV-coinfected patients: a systematic review and meta-analysis. Clin Infect Dis. 2010;50(9):1288-99. | 75 |
| 70 | Fox GJ, Barry SE, Britton WJ, Marks GB. Contact investigation for tuberculosis: a systematic review and meta-analysis. Eur Respir J. 2013;41(1):140-56. | 74 |
| 71 | Tiemersma EW, van der Werf MJ, Borgdorff MW, Williams BG, Nagelkerke NJ. Natural history of tuberculosis: duration and fatality of untreated pulmonary tuberculosis in HIV negative patients: a systematic review. PLoS One. 2011;6(4):e17601. | 74 |
| 72 | Perez-Guzman C, Vargas MH, Torres-Cruz A, Villarreal-Velarde H. Does aging modify pulmonary tuberculosis?: A meta-analytical review. Chest. 1999;116(4):961-7. | 73 |
| 73 | Rehm J, Samokhvalov AV, Neuman MG, Room R, Parry C, Lonnroth K, et al. The association between alcohol use, alcohol use disorders and tuberculosis (TB). A systematic review. BMC Public Health. 2009;9:450. | 73 |
| 74 | Pacheco AG, Cardoso CC, Moraes MO. IFNG +874T/A, IL10 -1082G/A and TNF -308G/A polymorphisms in association with tuberculosis susceptibility: a meta-analysis study. Hum Genet. 2008;123(5):477-84. | 72 |
| 75 | Machingaidze S, Wiysonge CS, Gonzalez-Angulo Y, Hatherill M, Moyo S, Hanekom W, et al. The utility of an interferon gamma release assay for diagnosis of latent tuberculosis infection and disease in children: a systematic review and meta-analysis. Pediatr Infect Dis J. 2011;30(8):694-700. | 72 |
| 76 | Gao L, Tao Y, Zhang L, Jin Q. Vitamin D receptor genetic polymorphisms and tuberculosis: updated systematic review and meta-analysis. Int J Tuberc Lung Dis. 2010;14(1):15-23. | 71 |
| 77 | Hirsch-Moverman Y, Daftary A, Franks J, Colson PW. Adherence to treatment for latent tuberculosis infection: systematic review of studies in the US and Canada. Int J Tuberc Lung Dis. 2008;12(11):1235-54. | 70 |
| 78 | Martin A, Portaels F, Palomino JC. Colorimetric redox-indicator methods for the rapid detection of multidrug resistance in Mycobacterium tuberculosis: a systematic review and meta-analysis. J Antimicrob Chemother. 2007;59(2):175-83. | 69 |
| 79 | Steingart KR, Flores LL, Dendukuri N, Schiller I, Laal S, Ramsay A, et al. Commercial serological tests for the diagnosis of active pulmonary and extrapulmonary tuberculosis: an updated systematic review and meta-analysis. PLoS Med. 2011;8(8):e1001062. | 69 |
| 80 | Li HT, Zhang TT, Zhou YQ, Huang QH, Huang J. SLC11A1 (formerly NRAMP1) gene polymorphisms and tuberculosis susceptibility: a meta-analysis. Int J Tuberc Lung Dis. 2006;10(1):3-12. | 69 |
| 81 | Baussano I, Williams BG, Nunn P, Beggiato M, Fedeli U, Scano F. Tuberculosis incidence in prisons: a systematic review. PLoS Med. 2010;7(12):e1000381. | 69 |
| 82 | Metcalfe JZ, Everett CK, Steingart KR, Cattamanchi A, Huang L, Hopewell PC, et al. Interferon-gamma release assays for active pulmonary tuberculosis diagnosis in adults in low- and middle-income countries: systematic review and meta-analysis. J Infect Dis. 2011;204 Suppl 4:S1120-9. | 68 |
| 83 | Kranzer K, Houben RM, Glynn JR, Bekker LG, Wood R, Lawn SD. Yield of HIV-associated tuberculosis during intensified case finding in resource-limited settings: a systematic review and meta-analysis. Lancet Infect Dis. 2010;10(2):93-102. | 68 |
| 84 | Menzies D, Benedetti A, Paydar A, Martin I, Royce S, Pai M, et al. Effect of duration and intermittency of rifampin on tuberculosis treatment outcomes: a systematic review and meta-analysis. PLoS Med. 2009;6(9):e1000146. | 67 |
| 85 | Minion J, Leung E, Menzies D, Pai M. Microscopic-observation drug susceptibility and thin layer agar assays for the detection of drug resistant tuberculosis: a systematic review and meta-analysis. Lancet Infect Dis. 2010;10(10):688-98. | 66 |
| 86 | Riquelme A, Calvo M, Salech F, Valderrama S, Pattillo A, Arellano M, et al. Value of adenosine deaminase (ADA) in ascitic fluid for the diagnosis of tuberculous peritonitis: a meta-analysis. J Clin Gastroenterol. 2006;40(8):705-10. | 66 |
| 87 | Menzies D, Benedetti A, Paydar A, Royce S, Madhukar P, Burman W, et al. Standardized treatment of active tuberculosis in patients with previous treatment and/or with mono-resistance to isoniazid: a systematic review and meta-analysis. PLoS Med. 2009;6(9):e1000150. | 65 |
| 88 | Minion J, Leung E, Talbot E, Dheda K, Pai M, Menzies D. Diagnosing tuberculosis with urine lipoarabinomannan: systematic review and meta-analysis. Eur Respir J. 2011;38(6):1398-405. | 64 |
| 89 | Suchindran S, Brouwer ES, Van Rie A. Is HIV infection a risk factor for multi-drug resistant tuberculosis? A systematic review. PLoS One. 2009;4(5):e5561. | 64 |
| 90 | Martin A, Panaiotov S, Portaels F, Hoffner S, Palomino JC, Angeby K. The nitrate reductase assay for the rapid detection of isoniazid and rifampicin resistance in Mycobacterium tuberculosis: a systematic review and meta-analysis. J Antimicrob Chemother. 2008;62(1):56-64. | 64 |
| 91 | Steingart KR, Henry M, Laal S, Hopewell PC, Ramsay A, Menzies D, et al. A systematic review of commercial serological antibody detection tests for the diagnosis of extrapulmonary tuberculosis. Thorax. 2007;62(10):911-8. | 61 |
| 92 | Lonnroth K, Williams BG, Cegielski P, Dye C. A consistent log-linear relationship between tuberculosis incidence and body mass index. Int J Epidemiol. 2010;39(1):149-55. | 60 |
| 93 | Pai M, Kalantri S, Pascopella L, Riley LW, Reingold AL. Bacteriophage-based assays for the rapid detection of rifampicin resistance in Mycobacterium tuberculosis: a meta-analysis. J Infect. 2005;51(3):175-87. | 59 |
| 94 | Georghiou SB, Magana M, Garfein RS, Catanzaro DG, Catanzaro A, Rodwell TC. Evaluation of genetic mutations associated with Mycobacterium tuberculosis resistance to amikacin, kanamycin and capreomycin: a systematic review. PLoS One. 2012;7(3):e33275. | 56 |
| 95 | Horne DJ, Royce SE, Gooze L, Narita M, Hopewell PC, Nahid P, et al. Sputum monitoring during tuberculosis treatment for predicting outcome: systematic review and meta-analysis. Lancet Infect Dis. 2010;10(6):387-94. | 56 |
| 96 | Ettehad D, Schaaf HS, Seddon JA, Cooke GS, Ford N. Treatment outcomes for children with multidrug-resistant tuberculosis: a systematic review and meta-analysis. Lancet Infect Dis. 2012;12(6):449-56. | 56 |
| 97 | Lewis SJ, Baker I, Davey Smith G. Meta-analysis of vitamin D receptor polymorphisms and pulmonary tuberculosis risk. Int J Tuberc Lung Dis. 2005;9(10):1174-7. | 55 |
| 98 | Mangtani P, Abubakar I, Ariti C, Beynon R, Pimpin L, Fine PE, et al. Protection by BCG vaccine against tuberculosis: a systematic review of randomized controlled trials. Clin Infect Dis. 2014;58(4):470-80. | 55 |
| 99 | Sun F, Chen Y, Xiang Y, Zhan S. Drug-metabolising enzyme polymorphisms and predisposition to anti-tuberculosis drug-induced liver injury: a meta-analysis. Int J Tuberc Lung Dis. 2008;12(9):994-1002. | 54 |
| 100 | Cox HS, Morrow M, Deutschmann PW. Long term efficacy of DOTS regimens for tuberculosis: systematic review. BMJ. 2008;336(7642):484-7 | 54 |
